# A Comprehensive Prognostic and Immunological Analysis of a Six-Gene Signature Associated With Glycolysis and Immune Response in Uveal Melanoma

**DOI:** 10.3389/fimmu.2021.738068

**Published:** 2021-09-22

**Authors:** Jun Liu, Jianjun Lu, Wenli Li

**Affiliations:** ^1^ Reproductive Medicine Center, Yue Bei People’s Hospital, Shantou University Medical College, Shaoguan, China; ^2^ Medical Research Center, Yue Bei People’s Hospital, Shantou University Medical College, Shaoguan, China; ^3^ Department of Medical Affairs, First Affiliated Hospital of Sun Yat-sen University, Guangzhou, China; ^4^ The Second School of Clinical Medicine, Southern Medical University, Guangzhou, China

**Keywords:** uveal melanoma, overall survival, glycolysis, immune response, gene signature

## Abstract

Uveal melanoma (UM) is a subtype of melanoma with poor prognosis. This study aimed to construct a new prognostic gene signature that can be used for survival prediction and risk stratification of UM patients. In this work, transcriptome data from the Molecular Signatures Database were used to identify the cancer hallmarks most relevant to the prognosis of UM patients. Weighted gene co-expression network, univariate least absolute contraction and selection operator (LASSO), and multivariate Cox regression analyses were used to construct the prognostic gene characteristics. Kaplan–Meier and receiver operating characteristic (ROC) curves were used to evaluate the survival predictive ability of the gene signature. The results showed that glycolysis and immune response were the main risk factors for overall survival (OS) in UM patients. Using univariate Cox regression analysis, 238 candidates related to the prognosis of UM patients were identified (*p* < 0.05). Using LASSO and multivariate Cox regression analyses, a six-gene signature including *ARPC1B*, *BTBD6*, *GUSB*, *KRTCAP2*, *RHBDD3*, and *SLC39A4* was constructed. Kaplan–Meier analysis of the UM cohort in the training set showed that patients with higher risk scores had worse OS (HR = 2.61, *p* < 0.001). The time-dependent ROC (t-ROC) curve showed that the risk score had good predictive efficiency for UM patients in the training set (AUC > 0.9). Besides, t-ROC analysis showed that the predictive ability of risk scores was significantly higher than that of other clinicopathological characteristics. Univariate and multivariate Cox regression analyses showed that risk score was an independent risk factor for OS in UM patients. The prognostic value of risk scores was further verified in two external UM cohorts (GSE22138 and GSE84976). Two-factor survival analysis showed that UM patients with high hypoxia or immune response scores and high risk scores had the worst prognosis. Moreover, a nomogram based on the six-gene signature was established for clinical practice. In addition, risk scores were related to the immune infiltration profiles. Taken together, this study identified a new prognostic six-gene signature related to glycolysis and immune response. This six-gene signature can not only be used for survival prediction and risk stratification but also may be a potential therapeutic target for UM patients.

## Introduction

Uveal melanoma (UM) is a rare and aggressive intraocular malignant tumor, and about 50% of patients are prone to liver metastases. The prognosis of metastatic UM is poor, and there is a lack of effective treatments. The average incidence per year is 5.1 per million people, which ranks first among intraocular tumors (1). About 90% of UMs are located in the choroid. Increased ocular melanocytes, choroidal nevi, and *BRCA1*-associated protein 1 mutations are considered to be risk factors for the occurrence of UM (2). The clinicopathological characteristics (such as the diameter of the basal tumor, ciliary body involvement, and scleral expansion), non-random chromosomal aberrations, gene mutations (such as *BAP1* and *SF3B1* mutations) are considered to be related to the prognosis of patients with UM (3–5). Recent studies have shown that percutaneous hepatic perfusion can control the progression of UM metastatic intrahepatic lesions (6). Meanwhile, early surgical treatment of UM liver metastases may help improve the progression-free survival (PFS) and overall survival (OS) in UM patients with liver metastases (7–11). Therefore, early diagnosis and surgical treatment are essential to improve the prognosis of patients with UM (12, 13).

Therefore, screening new survival predictors is of great significance for guiding the diagnosis and treatments of UM. However, on the one hand, the predictive ability of traditional clinicopathological features such as pathological staging has been shown to be insufficient. On the other hand, previous studies have used global gene expression data to build signatures that are likely to bring gene sets from different biological processes, masking the key biological features driving prognosis. Therefore, our approach was to start with the biological processes relevant in UM and construct signatures around the same to create a gene signature with fewer genes than those constructed in earlier studies.

Previous studies have shown that cancer hallmarks, such as glycolysis and immune response, are associated with the prognosis of UM patients (14, 15). As is known, immunotherapy can induce an immune response in certain cancers to help control cancer progression. However, due to reasons such as the lack of *BRAF* mutations and loss of *BAP1* expression, the response rate of UM to immune checkpoint blockade is very low (16). Studies have shown that the high density of tumor-associated macrophages and infiltrating T lymphocytes in UM is related to the poor prognosis of UM patients (17). Therefore, although the mutation burden of UM is low, it does have immunogenicity. This is of great significance to the development of immunotherapy (18). Therefore, the prognostic gene signature constructed using the gene sets of immune response may be more related to the immune cell infiltration status and help discover potential targets for immunotherapy in UM.

In this study, we extracted an UM cohort from The Cancer Genome Atlas (TCGA), screened out the most important prognostic genes related to the two cancer hallmarks, glycolysis and immune response, and established a UM survival prediction gene signature. Subsequently, the prognostic value of the risk model based on the gene signature was verified in two independent UM validation sets from the Gene Expression Omnibus (GEO) database. Next, the prognostic values of the risk models and cancer markers (such as glycolysis and immune response) were further analyzed. A time-dependent receiver operating characteristic (t-ROC) curve was used to verify the predictive accuracy of the gene signature. In addition, the correlation between the risk model and the tumor immune microenvironment (TIME) was explored. In conclusion, this study constructed a new prognostic gene signature related to cancer hallmarks, including glycolysis and immune response, for patients with UM. This gene signature could help develop individualized treatment plans for patients with UM and may be expected to serve as a potential prognostic biomarker for UM.

## Material and Methods

### Dataset Preparation and Data Processing

The UM dataset containing the messenger RNA (mRNA) expression profiles and clinical information of 80 UM patients obtained from TCGA database (http://cancergenome.nih.gov/) was used as the training dataset for establishing the prognostic model. The validation datasets GSE22138 and GSE84976, with the UM-mRNA expression profiles and clinical information downloaded from the GEO (http://www.ncbi.nlm.nih.gov/geo/) database, included 63 and 28 UM patients, respectively. The two above-mentioned databases are publicly available. Therefore, this study did not require the approval of the local ethics committee.

### Candidate Selection and Gene Signature Establishment

Using the transcriptome profiling data and hallmark gene sets from the Molecular Signatures Database (MSigDB) and the single-sample gene set enrichment analysis (ssGSEA) (R package “gsva”), the performance of the cancer hallmarks in the training set was quantified for univariate Cox proportional hazards (Cox-PH) regression analysis to evaluate the significance of various cancer hallmarks in UM (R package “survival”) (19, 20). Weighted gene co-expression network analysis (WGCNA) was used to construct a scale-free co-expression network (R package “wgcna”) to identify the gene module most relevant to glycolysis and immune response based on data from the transcriptome analysis and the ssGSEA scores (21). Meanwhile, the correlations between individual genes and the ssGSEA scores of the cancer hallmarks were quantified by gene significance (GS); the correlations between module characteristic genes and the gene expression profiles were represented by module members (MM). Using the *p*-value threshold of GS <0.0001 and the significance threshold of univariate Cox regression with *p* < 0.01, 238 genes extracted from the module as most related to glycolysis and immune response were screened as candidates. Next, the least absolute shrinkage and selection operator (LASSO) Cox regression model was used to further screen the most reliable prognostic biomarkers (22). Specifically, univariate Cox regression analysis was conducted to identify genes related to OS, and then LASSO Cox regression was applied to further narrow the scope of the UM marker genes. In terms of the calculation principle, LASSO regression can not only solve the overfitting problem but also extract useful features by directly reducing some repeated unnecessary parameters to zero in the parameter reduction process. Subsequently, multiple Cox regression analysis was performed to evaluate whether marker genes can be used as independent prognostic factors for patient survival. Next, WGCNA was used to construct a gene co-expression network. The risk model related to glycolysis and immune response was established by including standardized gene expression values weighted by its LASSO Cox coefficient. The formula is as follows:


$$\text{Risk score}=\sum_{i}\text{Coefficient}\;({\text{mRNA}}_{i})*\text{Expression}\;(\text{mRNAi})$$

### Survival Analysis Based on the Risk Model

Taking the median risk score as the cutoff value, we divided the UM patients into a high-rick and a low-risk group. Subsequently, the prognosis of the two groups was compared. The ROC curve was used to evaluate the prediction accuracy of the risk model. Moreover, a two-factor survival analysis combining the risk scores and cancer hallmarks (such as glycolysis and immune response) was conducted in the training set and the validation set GSE22138 to comprehensively evaluate the impact of risk scores and cancer hallmarks on the prognosis of UM patients.

### Robustness of the Six-Gene Signature in UM

To confirm the prognostic values of the genes from another perspective, six genes were randomly selected from the UM transcriptome data for evaluation of their prognostic performance. This process was repeated 1,000 times to increase statistical power. In addition, previous studies have constructed some gene signatures that can be used to predict the survival of UM patients. For example, a 10-gene signature constructed to predict the survival of UM patients showed good survival predictive ability (23). Therefore, in order to further explore the prognostic significance of the gene signature constructed in this study, we compared the predictive power of five gene signatures for UM patients.

### Prognostic Value of the Gene Signature in Other Tumors

In order to fully understand the prognostic value of our gene signature in tumors, we evaluated its ability to predict survival in other tumors. As is known, skin melanoma (SM) is another subtype of melanoma and hepatocellular carcinoma (HCC) is a visceral tumor. Therefore, SM and HCC were used as the cancer types to evaluate the survival predictive ability of our gene signature in tumors other than UM.

### Establishment and Evaluation of Survival Prediction Nomogram for UM Patients

Nomogram is an effective method for predicting the prognosis of cancer patients by simplifying complex statistical prediction models into contour maps that assess the probability of OS of individual patients (24). In this study, we constructed a nomogram based on the six-gene signature to evaluate the probability of OS in UM patients at 1, 3, and 5 years. Meanwhile, the predicted probability of the nomogram is compared with the observed actual probability through a calibration curve in order to verify the accuracy of the nomogram. An overlap with the reference line indicates that the model is accurate.

### Correlation Analysis Between Risk Scores and TIME

The immune-related complex environment for the survival and development of tumor cells is described as the tumor microenvironment. In this study, four analysis methods, namely, TIMER, CIBERSORT, quanTIseq, and xCELL, were applied to analyze the correlations between the risk scores and immune cell infiltration status. Heat maps and bar graphs were drawn to compare the immune infiltration levels of the various immune cells in the high- and low-risk groups. Furthermore, we performed a two-factor survival analysis combining the immune cell infiltration levels and the risk scores. In addition, this study explored the correlation between the risk scores and some immune checkpoint molecules.

### Bioinformatics and Statistical Analysis

GSEA using the glycolysis and immune response genome from MSigDB was performed to verify the glycolysis and immune response status of the high-risk group (25). IBM SPSS Statistics 20 (IBM Corp., Armonk, NY, USA) and R software (version 3.5.2; http://www.r-project.org) were used to analyze the data and draw charts. The *Z*-score method was utilized to normalize the ssGSEA scores and the risk scores based on the gene signature. The Kaplan–Meier method was employed to draw the survival curve and the log-rank test used to assess differences between groups. The Cox-PH regression model was used to evaluate the importance of each parameter to OS. The t-ROC analysis was performed to measure the predictive power of the risk model (R package “survival-ROC”) (26). The areas under the ROC curve of the various variables at different time points (AUC-t) were compared. The “wilcox.test” function was applied to compare the risk scores of the two groups; the “kruskal.test” function was used to compare the risk scores of the different pathological stages.

## Results

### Schematic Diagram of the Study Design

**Figure 1:**
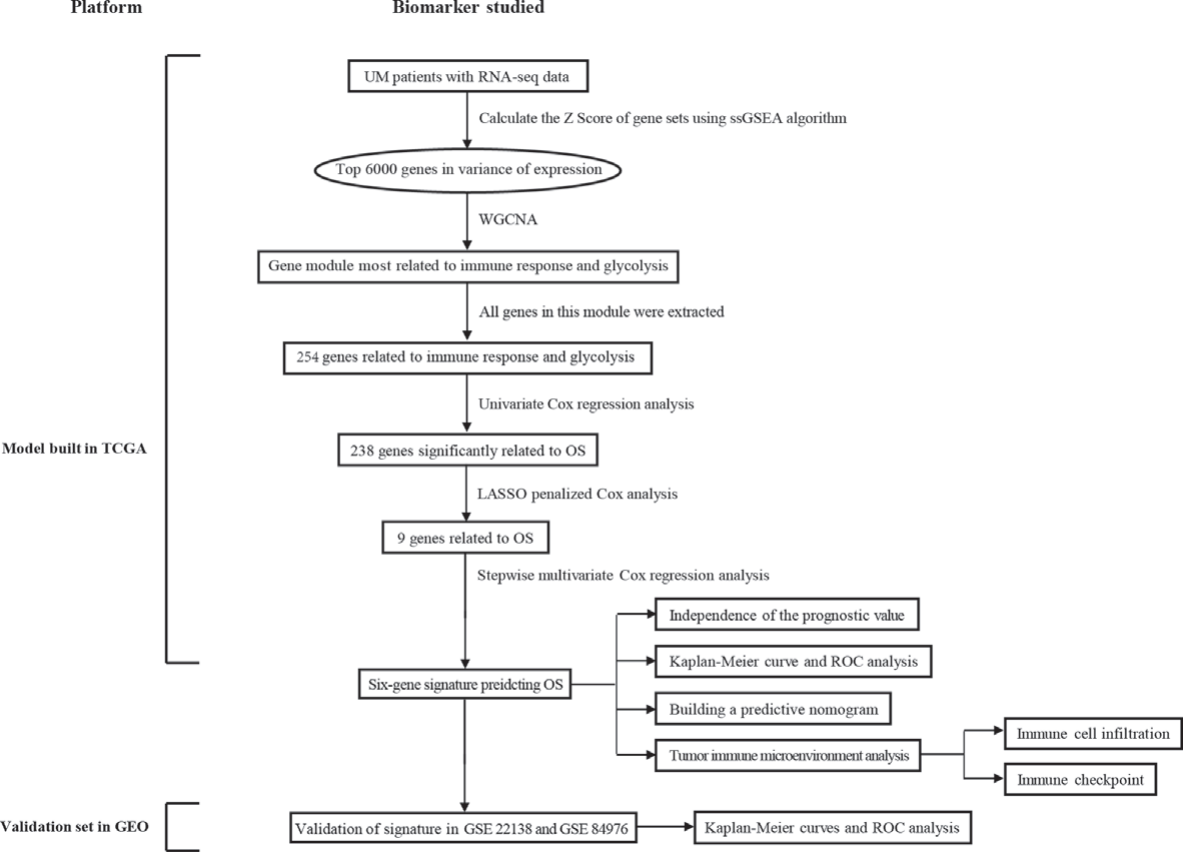
Overall flowchart of this study. *LASSO*, least absolute shrinkage and selection operator; *UM*, uveal melanoma; *ssGSEA*, single-sample gene set enrichment analysis; *ROC*, receiver operating characteristic; *WGCNA*, weighted gene co-expression network analysis.

**Figure 1** displays the flowchart of the entire work. The figure shows the detailed construction process of the prediction model in predicting the OS of patients with UM. At the start, glycolysis and immune response are identified as the primary cancer hallmarks for survival in UM. Next, WGCNA, univariate and multivariate Cox regression analyses, and the LASSO algorithm were employed to identify the hub genes related to glycolysis and immune response. Furthermore, the identified hub genes were used to establish the risk model predicting the OS of UM patients. Subsequently, the prognostic value of the risk model was evaluated in the training set and in two independent validation sets. Information of the patients in TCGA and the GEO cohorts is shown in **Table 1**.

**Table 1:** Baseline demographic and clinical information in The Cancer Genome Atlas (TCGA) and the Gene Expression Omnibus (GEO) cohorts.

Clinical characteristics		Total	%
TCGA		80	
Status	Alive	57	71.25
	Dead	23	28.75
Age (years)	<60	36	45
	≥60	44	55
Sex	Female	35	43.75
	Male	45	56.25
T	T2	14	17.5
	T3	32	40
	T4	34	42.5
Stage	Stage II	36	45
	Stage III	40	50
	Stage IV	4	5
GSE22138		63	
Status	Alive	28	44.44
	Dead	35	55.56
Age (years)	<60	28	44.44
	≥60	35	55.56
Sex	Female	24	38.1
	Male	39	61.9
GSE84976		28	
Status	Alive	12	42.86
	Dead	16	57.14
Age (years)	<60	12	42.86
	≥60	16	57.14

### Glycolysis and Immune Response Were Identified as the Primary Risk Factors for OS of UM Patients

**Figure 2:**
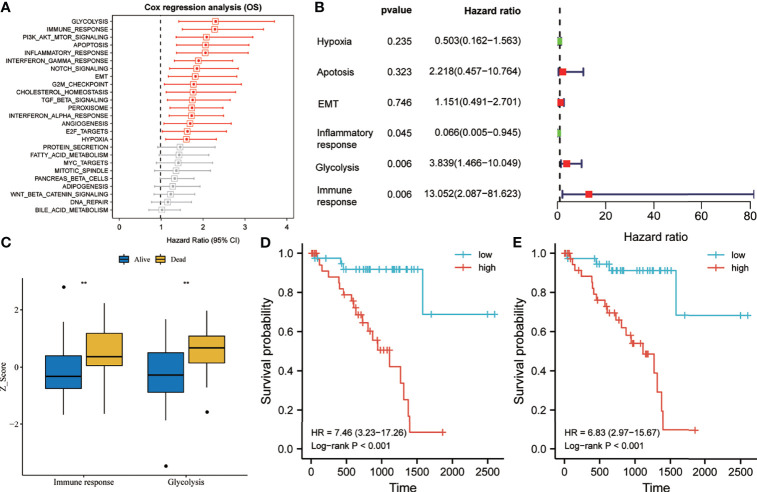
Glycolysis and immune response are identified as the primary risk factors for survival in uveal melanoma (UM) patients. **(A)** Univariate Cox regression analysis showed that glycolysis and immune response were the main hallmarks of cancer that affect the overall survival (OS) of UM patients. **(B)** Multivariate Cox regression analysis showed that glycolysis and immune response were independent risk factors that affect the OS of UM patients. **(C)** The glycolysis and immune response *Z*-scores of patients who died during follow-up were significantly higher than those of patients who are still alive. **(D, E)** Kaplan–Meier analysis suggested that patients with higher glycolysis and immune response *Z*-scores exhibited poorer OS. *EMT*, epithelial–mesenchymal transition; *Pl3/Akt/mTOR*, phosphatidylinositol–3-kinase-Akt–mammalian target of rapamycin; *TGF*, transforming growth factor. **p < 0.01.

According to the ssGSEA scores and survival information of the cancer hallmarks in the training set, the Cox coefficient of each cancer hallmark was calculated and sorted. Univariate analysis suggested that, compared with the other cancer hallmarks such as PI3K/AKT/mTOR signaling, apoptosis, EMT, G2M checkpoint, TGF-beta signaling, angiogenesis, and hypoxia, glycolysis and immune response were the most significant risk factors affecting the survival of UM patients (**Figure 2A**). Multivariate analysis suggested that glycolysis and immune response were independent prognostic factors for OS of UM patients (**Figure 2B**). As shown in **Figure 2C**, the glycolysis and immune response *Z*-scores of UM patients who died during follow-up were significantly higher than those of patients who are still alive. Subsequently, taking the median of the risk score as the cutoff value, we divided the 80 UM patients in the training set into a high-risk group and a low-risk group. Survival analysis suggested that the OS rate of the group with high glycolysis *Z*-scores was lower than that of the group with low glycolysis *Z*-scores [hazard ratio (HR) = 7.46, *p* < 0.001] (**Figure 2D**). Meanwhile, the OS rate of the group with high immune response *Z*-scores was lower than that of the group with low immune response *Z*-scores (HR = 6.83, *p* < 0.001) (**Figure 2E**).

### Establishment of Prognostic Gene Signature Related to Glycolysis and Immune Response

**Figure 3:**
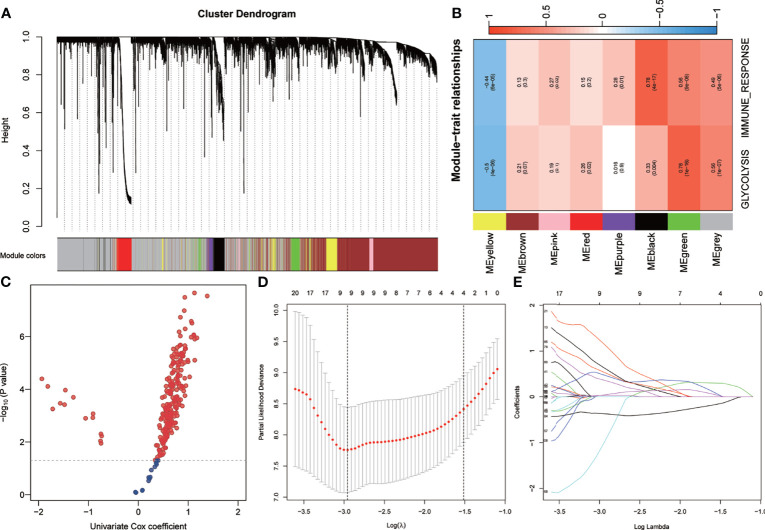
Establishment of genetic signatures related to glycolysis and immune response. **(A)** The variance of the expression value of each gene in the training set was calculated, and then the top 6,000 genes in the variance ranking were used to construct the WGCNA network. Seven non-gray modules were identified. **(B)** The green module depicting the highest correlation (*r* > 0.5, *p* < 0.0001) was considered the most correlated with glycolysis and immune response. **(C)** Using univariate Cox regression analysis, 238 prognostic-related candidate genes were extracted from the key genes from the green module. **(D, E)** The LASSO Cox regression model was used to identify the most robust biomarkers, and multivariate Cox stepwise regression was applied to construct a prognostic model. *LASSO*, minimum absolute shrinkage and selection operator; *WGCNA*, weighted gene co-expression network analysis.

WGCNA was performed using the whole-transcriptome analysis data and the ssGSEA *Z*-scores of glycolysis and immune response in the training set. A total of seven non-gray modules were generated (**Figure 3A**). Among these modules, the green module had the highest correlation with glycolysis and immune response (*r* > 0.5, *p* < 0.0001) (**Figure 3B**). With a *p*-value threshold for GS of <0.0001, the hub genes extracted from the green module were used for univariate Cox regression analysis. With a *p*-value threshold for univariate Cox regression of <0.01, 238 candidates with prognostic values were screened out (**Figure 3C**). Subsequently, LASSO Cox regression was performed to determine the most robust prognostic biomarkers. By applying a 10-fold cross-validation to overcome overfitting, nine variables were identified (**Figures 3D, E**). With multivariate Cox stepwise regression analysis, six hub genes were identified for constructing a prognostic risk model for UM patients: risk score = 3.80 * *ARPC1B* + 4.02 * *BTBD6* + 1.02 * *GUSB* + 2.10 * *KRTCAP2* − 6.65 * *RHBDD3* + 2.25 * *SLC39A4*.

### Risk Score Is an Independent Risk Factor for OS of Patients with UM in the Training Set

**Figure 4:**
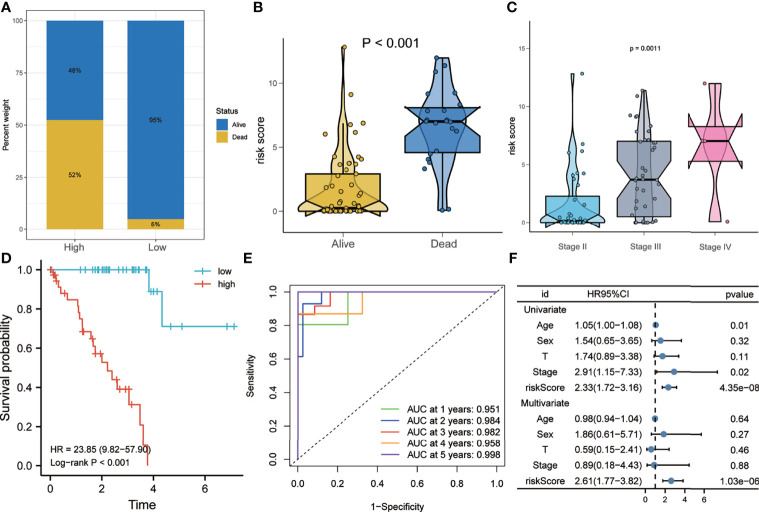
Risk score predicts poor survival in the training set. **(A)** Patients in the high-risk group had a higher mortality rate. **(B)** Patients who died during follow-up had higher risk scores. **(C)** More advanced tumor stages had higher risk scores. **(D)** Kaplan–Meier analysis showed that patients with higher risk scores had worse overall survival. **(E)** The receiver operating characteristic (ROC) curve showed that the risk score exhibited good predictive efficiency for OS (AUC > 0.9). **(F)** Univariate and multivariate Cox regression analyses showed that risk score was an independent risk factor for OS. *AUC*, area under the ROC curve; *HR*, hazard ratio; *OS*, overall survival.

As shown in **Figure 4A**, the proportion of patients who died during follow-up in the high-risk group was higher than that in the low-risk group in the training set. **Figure 4B** shows that the risk scores of patients who died during follow-up were significantly higher than those of patients who are still alive. In addition, patients with more advanced pathological stages had higher risk scores (**Figure 4C**). Kaplan–Meier analysis showed that patients with higher risk scores had a worse prognosis than did patients with lower risk scores (HR = 23.85, *p* < 0.001) (**Figure 4D**). The ROC curve showed that the AUCs of risk scores predicting the 1-, 2-, 3-, 4-, and 5-year survival rates were above 0.9, suggesting that risk score was a good model for predicting the survival of UM patients (**Figure 4E**). At the same time, multivariate Cox regression analysis suggested that, among the various clinicopathological variables in the training set, risk score was an independent risk factor for OS (HR = 2.61, *p* < 0.001) (**Figure 4F**).

### Risk Score Is a Risk Factor for Disease Progression in the Training Set

**Figure 5:**
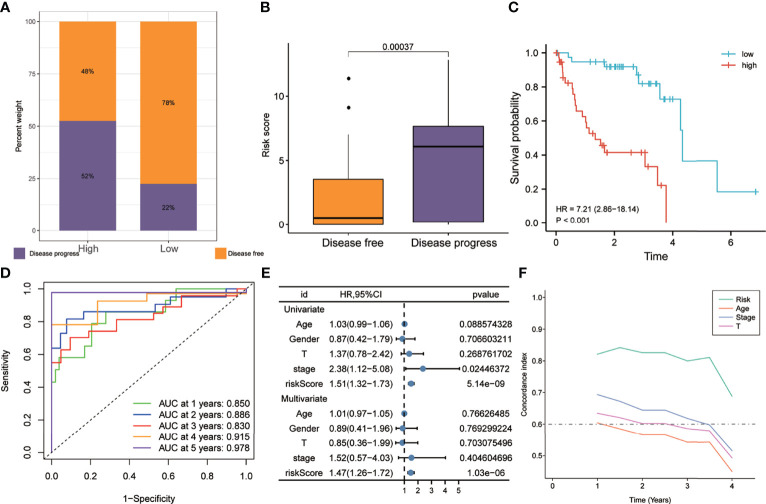
Risk score predicts a higher rate of disease progression in the training set. **(A)** The proportion of patients with disease progression in the high-risk group was higher. **(B)** Patients with disease progression had higher risk scores. **(C)** Patients in the high-risk group exhibited worse PFS. **(D)** The receiver operating characteristic (ROC) curve showed good predictive accuracy of the risk score on PFS (AUC > 0.8). **(E)** Univariate and multivariate Cox regression analyses showed that risk score was an independent risk factor for PFS. **(F)** The t-ROC analysis showed that the survival predictive power of the risk model was significantly better than that of other clinical features. *AUC*, area under the ROC curve; *HR*, risk ratio; *PFS*, progression-free survival; *t-ROC*, time-dependent receiver operating characteristics.

As shown in **Figure 5A**, the proportion of patients with disease progression in the high-risk group was higher than that in the low-risk group in the training set. **Figure 5B** shows that the risk scores of patients with disease progression during follow-up were significantly higher than those of disease-free patients. Kaplan–Meier analysis showed that the PFS of the high-risk group was worse than that of the low-risk group (HR = 7.21, *p* < 0.001) (**Figure 5C**). The ROC curve showed that the AUCs of risk scores predicting the 1-, 2-, 3-, 4-, and 5-year survival rates were above 0.8, suggesting that risk score was a good model for predicting PFS in UM patients (**Figure 5D**). Meanwhile, multivariate Cox regression analysis suggested that, among the various clinicopathological variables in the training cohort, risk score was an independent risk factor for PFS (HR = 1.47, *p* < 0.001) (**Figure 5E**). In addition, the t-ROC analysis suggested that risk score was an accurate indicator for predicting PFS (**Figure 5F**).

**Figure 6:**
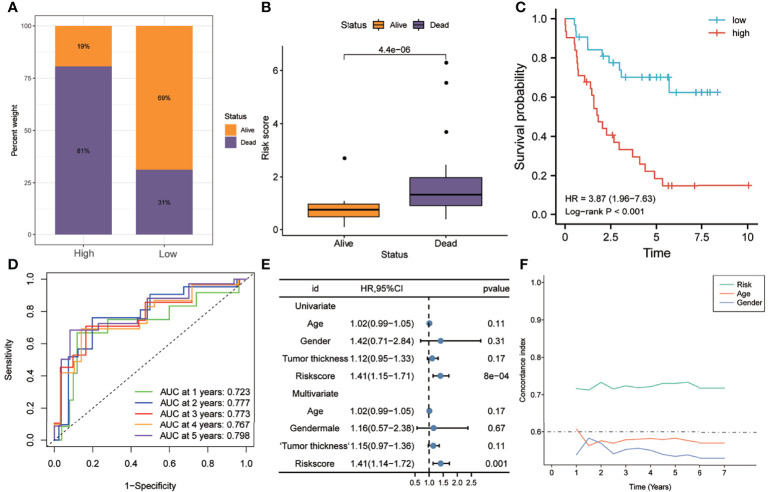
Validation of the risk model in the GSE22138 dataset. **(A)** Patients in the high-risk group had higher mortality rates. **(B)** The risk scores of patients who died during follow-up were higher. **(C)** Kaplan–Meier analysis showed that patients with higher risk scores had worse overall survival. **(D)** The ROC curve showed good predictive accuracy of the risk score for OS (AUC > 0.7). **(E)** Univariate and multivariate Cox regression analyses showed that risk score was an independent risk factor for OS. **(F)** The t-ROC analysis showed that the survival predictive power of the risk model was significantly better than that of other clinical features. *AUC*, area under the ROC curve; *HR*, hazard ratio; *OS*, overall survival; *t-ROC*, time-dependent receiver operating characteristics.

In order to further verify the robustness of the risk model in predicting the survival of UM patients, it was validated in independent external UM cohorts. As shown in **Figure 6A**, the proportion of patients who died in the high-risk group was higher than that in the low-risk group. **Figure 6B** shows that the risk scores of patients who died were significantly higher than those of patients who are still alive (*p* < 0.0001). Kaplan–Meier analysis suggested that the OS of the high-risk group was worse than that of the low-risk group (HR = 3.87, *p* < 0.001) (**Figure 6C**). The ROC curve showed that the AUCs of risk scores predicting the 1-, 2-, 3-, 4-, and 5-year survival rates were above 0.7, suggesting that risk score was a good model for predicting the OS of UM patients (**Figure 6D**). Multivariate Cox regression analysis suggested that, among the various clinicopathological variables, risk score was an independent risk factor for OS (HR = 1.41, *p* < 0.001) (**Figure 6E**). In addition, t-ROC analysis suggested that risk score was an accurate indicator for predicting the OS of UM patients (**Figure 6F**).

**Figure 7:**
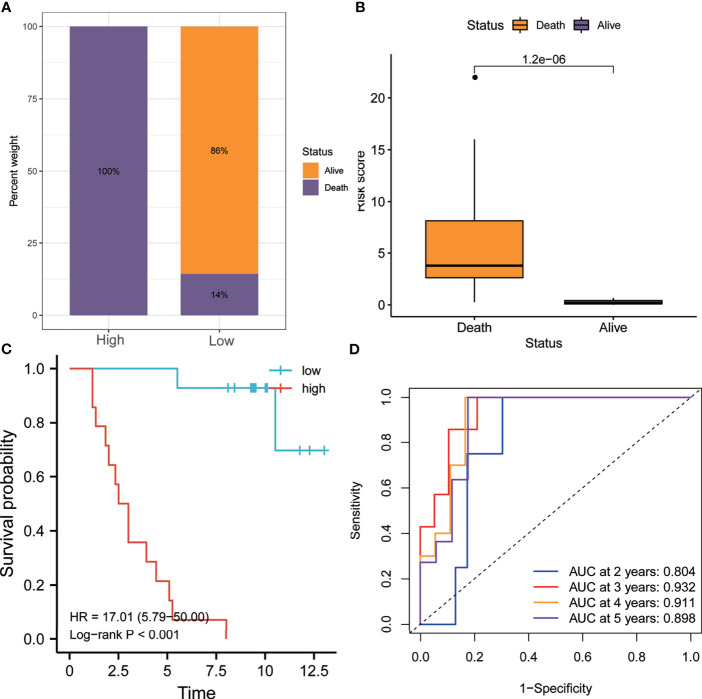
Validation of the risk model in the GSE84976 dataset. **(A)** Patients in the high-risk group had higher mortality rates. **(B)** The risk scores of patients who died during follow-up were higher. **(C)** Kaplan–Meier analysis showed that patients with higher risk scores had worse overall survival. **(D)** The receiver operating characteristic (ROC) curve showed good predictive accuracy of the risk score for OS (AUC > 0.8). *AUC*, area under the ROC curve; *HR*, hazard ratio; *OS*, overall survival.

Similarly, as shown in **Figure 7A**, the proportion of patients who died in the high-risk group was higher than that in the low-risk group. **Figure 7B** shows that the risk score of patients who died during follow-up was significantly higher than that of patients who are still alive (*p* < 0.0001). Kaplan–Meier analysis showed that the OS of the high-risk group was worse than that of the low-risk group (HR = 17.01, *p* < 0.001) (**Figure 7C**). In addition, the ROC curve showed that the AUCs of risk scores for predicting the 1-, 2-, 3-, 4-, and 5-year survival rates were all above 0.8, suggesting that risk score was a good model for predicting the OS of UM patients (**Figure 7D**).

### Correlation of Risk Scores With Cancer Hallmarks, Including Glycolysis and Immune Response, and Corresponding Two-Factor Survival Analysis

**Figure 8:**
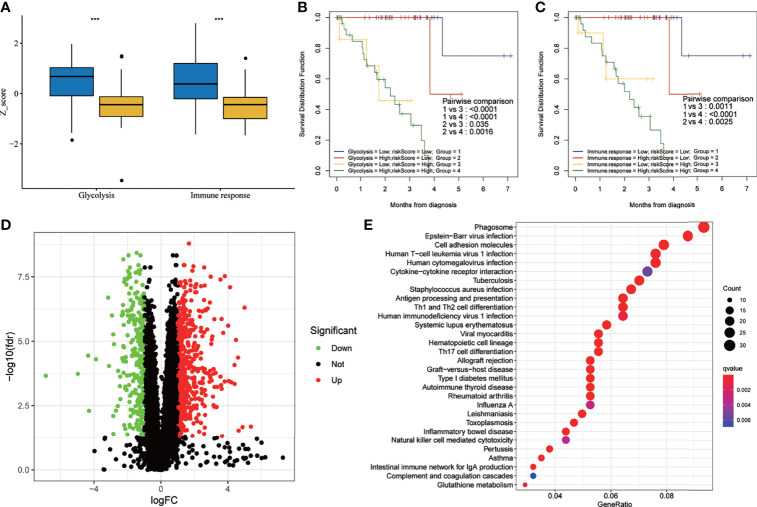
Two-factor survival analysis combining the cancer hallmarks and risk scores in the training set. **(A)** The glycolysis and immune response *Z*-scores in the high-risk group were higher than those of the low-risk group. **(B)** Two-factor survival analysis combining glycolysis and risk scores suggested that high glycolysis *Z*-scores and high risk scores predicted worse prognosis. **(C)** Two-factor survival analysis combining immune response and risk scores suggested that high immune response *Z*-scores and high risk scores predicted worse prognosis. **(D)** Volcano map showing the DEGs between the high- and low-risk groups in the training set (with |log2FC| > 1, FDR < 0.05 as the cutoff value). **(E)** Kyoto Encyclopedia of Genes and Genomes (KEGG) analysis of DEGs. *FDR*, false discovery rate; *DEGs*, differentially expressed genes. ***p < 0.001.

The results of the two-factor survival analysis combining the risk scores and cancer hallmarks in the training set are shown in **Figure 8**. The high-risk group had higher glycolytic and immune response *Z*-scores (**Figure 8A**). UM patients with low risk scores and low glycolytic or immune response *Z*-scores showed the best OS, while those with high risk scores and high glycolytic or immune response *Z*-scores showed a worse prognosis (**Figures 8B, C**). Using differentially expressed gene (DEG) analysis, a total of 814 DEGs, including 259 downregulated and 555 upregulated genes, were identified [with |log2FC| > 1, false discovery rate (FDR) <0.05 as the cutoff value] (**Figure 8D**). In addition, the Kyoto Encyclopedia of Genes and Genomes (KEGG) analysis showed that the main enrichment pathways of these DEGs included phagosome, Epstein–Barr virus infection, cell adhesion molecules, human T-cell leukemia virus 1 infection, human cytomegalovirus infection, cytokine–cytokine receptor interaction, tuberculosis, *Staphylococcus aureus* infection, antigen processing and presentation, and Th1 and Th2 cell differentiation (**Figure 8E**). Meanwhile, the results of the two-factor survival analysis combining the risk scores and cancer hallmarks in the validation set (GSE22138) are shown in **Figure S1**. The high-risk group had higher immune response and glycolytic *Z*-scores (**Figure S1A**). UM patients with low risk scores and low immune response or glycolytic *Z*-scores showed the best OS, while those with high risk scores and high immune response or glycolytic *Z*-scores showed a worse prognosis (**Figures S1B, C**).

### The Six-Gene Signature Is a Good Predictive Model With Fewer Genes

In order to confirm the predictive ability of the six-gene signature constructed in this study from another perspective, six genes were randomly selected from the UM transcriptome data for evaluation of the prognostic performance. As shown in **Table S1**, 1,000 randomly selected six-gene combinations failed to predict the survival of UM patients. These results further confirmed the predictive ability of the six-gene signature. In addition, we considered that a comparison of the predictive abilities of various gene signatures for UM patients could help to further explore the prognostic values of these gene signatures. Therefore, the predictive abilities of five gene signatures were compared. The five included signatures are defined as follows: 1) the six-gene signature constructed in this study; 2) the 10-gene signature (*SIRT3*, *HMCES*, *SLC44A3*, *TCTN1*, *STPG1*, *POMGNT2*, *RNF208*, *ANXA2P2*, *ULBP1*, and *CA12*) constructed by Luo et al. (23); 3) the 16-gene signature constructed by combining the six-gene and 10-gene signatures; 4) the five-gene signature (*ANXA2P2*, *CA12*, *HMCES*, *SIRT3*, and *SLC44A3*) constructed by performing stepwise multifactor Cox regression analysis on the 10-gene signature; and 5) the random six-gene signature constructed by randomly selecting six genes from the UM transcriptome data. As shown in **Figures S2A, B**, the t-ROC analysis and the concordance index (C-index) analysis showed that the 6-gene, 10-gene, 16-gene, and 5-gene signatures had good survival predictive capabilities (AUC > 0.85). The prediction results of these four gene signatures were well consistent with the survival results actually observed in UM. In addition, the random six-gene signature failed to predict survival in UM (AUC < 0.57).

### The Survival Prediction Ability of the Six-Gene Signature in UM Was Much Better Than That in SM and HCC

In order to further explore the prognostic value of the six-gene signature in tumors, we evaluated its predictive ability for OS in SM and HCC. Kaplan–Meier analysis showed that SM patients with higher risk scores had a worse prognosis than those with lower risk scores (HR = 1.56, *p* = 0.001) (**Figure S2C**). However, the ROC curves showed that the predictive ability of the six-gene signature in SM was low (AUC < 0.63) (**Figure S2D**). Similarly, HCC patients with higher risk scores had a worse prognosis than those with lower risk scores (HR = 1.74, *p* = 0.002) (**Figure S2E**). Also, the predictive ability of the six-gene signature in HCC was low (AUC < 0.66) (**Figure S2F**). These results indicate that the survival predictive ability of the six-gene signature in UM was much better than that in SM and HCC.

### Building a Nomogram to Predict OS in UM Patients

We constructed a nomogram for clinical practice based on the six-gene signature (**Figure S3A**). Then, a calibration curve was created to verify the predictive ability of the nomogram (**Figure S3B**). The calibration chart showed that the predicted 1-, 3-, and 5-year survival probabilities were in good agreement with the actual observations.

### Correlation Analysis Between Risk Score and the Immune Cell Infiltration Profiles

**Figure 9:**
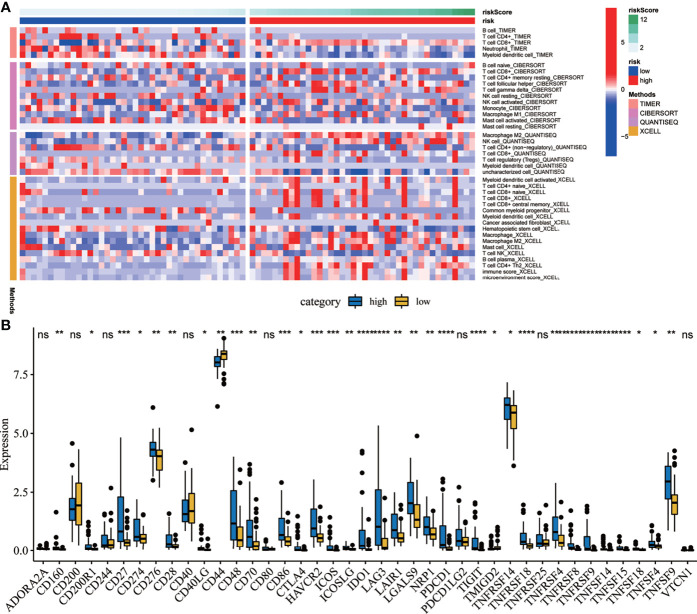
Correlation analysis between the risk scores and the immune microenvironment. **(A)** Heat map showing the correlations between the risk scores and immune cell infiltration using four methods. **(B)** Correlation analysis between the risk scores and several immune checkpoint molecules. *P < 0.05. **P < 0.01. ***P < 0.001. ****P < 0.0001. ns, no significance.

A heat map was drawn to detect the correlations between the risk scores and the levels of immune cell infiltration. Four methods were applied in this study: TIMER, CIBERSORT, quanTIseq, and xCELL. As shown in **Figure 9A**, the results of TIMER suggest that the infiltration levels of B cells and CD4^+^ T cells were higher in the low-risk group. The results from CIBERSORT showed that the infiltration levels of B cells, CD4^+^ T cells, and monocytes were higher in the low-risk group. Meanwhile, the results from quanTIseq showed that the immune infiltration levels of M2 macrophages were higher in the high-risk group. The results of xCELL suggested that the immune infiltration levels of myeloid dendritic cells were higher in the high-risk group. Moreover, immune checkpoints are sensitive molecules expressed by immune cells that can regulate immune activation. Therefore, we explored the correlations between the risk scores and the expression levels of various immune checkpoint molecules. The results suggested that the expression levels of immune checkpoint molecules such as *CD27*, *CD48*, *CD86*, *HAVCR2*, *ICOS*, and indoleamine 2,3-dioxygenase 1 (*IDO1*) were significantly correlated (**Figure 9B**).

### UM Patients With High Risk Scores and Low Immune Cell Scores Showed Poor OS

Immune cell infiltration in the tumor microenvironment affects the prognosis of tumor patients. Therefore, this study explored the correlations between the risk scores and the infiltration status of various immune cells. The results from TIMER suggested that the risk score was significantly related to the infiltration level of immune cells such as B cells, CD8 T cells, and myeloid dendritic cells (**Figure S4A**). The results from CIBERSORT suggested that the risk score was significantly correlated with the infiltration level of immune cells such as CD8 T cells, monocytes, and activated mast cells (**Figure S4B**). The results of quanTIseq suggested that the risk score was significantly related to the infiltration levels of immune cells such as M2 macrophages, natural killer (NK) cells, regulatory T cells, CD4 T cells, CD8 T cells, and myeloid dendritic cells (**Figure S4C**). The results of xCELL suggested that the risk score was significantly related to the infiltration levels of immune cells such as central memory CD8^+^ T cells, common myeloid progenitors, macrophages, M2 macrophages, NK cells, and Th2 CD4 T cells (**Figure S4D**). The two-factor survival analysis combining the immune cell scores and risk scores showed that UM patients with low risk scores and high immune cell scores showed better OS, while those with high risk scores and low immune cell scores showed poor OS (**Figures S4E–H**).

## Discussion

UM is a malignant tumor with poor prognosis. Early diagnosis and treatment are of great significance for improving the survival of patients with UM. However, there is currently a lack of accurate prognostic biomarkers for UM. Previous studies have shown that cancer hallmarks such as glycolysis and immune response are related to the prognosis of UM patients (14, 15). Therefore, this study focused on constructing a gene signature with prognostic values related to glycolysis and immune response. Firstly, using ssGSEA and Cox-PH regression models, glycolysis and immune response were identified as the cancer hallmarks most relevant to the OS of UM patients. Then, using WGCNA, the gene module most related to glycolysis and immune response was identified. Next, using univariate, LASSO, and multivariate regression analyses, hub genes (including *ARPC1B*, *BTBD6*, *GUSB*, *KRTCAP2*, *RHBDD3*, and *SLC39A4*) with prognostic values from the gene module were identified. Finally, the survival analysis results in the training and validation sets confirmed that this six-gene signature is an independent prognostic predictor of OS for patients with UM. Furthermore, we established a nomogram based on the six-gene signature that can be used in clinical practice.

As is known, the occurrence and progression of many human cancers are related to cancer hallmarks such as immune response, and immunotherapy has curative effects on certain cancers (27–29). It has been reported that immune cells such as CD8^+^ T cells, monocytes, memory CD4^+^ T cells, and mast cells are significantly related to the OS of UM patients (30). Moreover, some immune-related gene signatures related to the survival of UM patients have been identified (15). Immune checkpoints such as TIGIT and IDO are considered as potential therapeutic targets for UM patients (31). However, the low immunogenicity of UM greatly limits the application of immunotherapy on UM (32–34). It is worth mentioning that recent studies have suggested that adoptively transferred tumor-infiltrating lymphocytes have shown curative effects on metastatic UM (27). In addition, PD-L1 is expressed in 10% of UM, and anti-PD-1-based treatment may bring clinical benefits to patients with metastatic UM (35, 36). Blood biomarkers such as lactate dehydrogenase (LDH), C-reactive protein (CRP), and relative eosinophil count (REC) can be used to predict the survival prognosis of patients treated with immune checkpoint blockade (37). Therefore, this study explored the correlation between this prognostic six-gene signature and immune cell infiltration. The results showed that the infiltration levels of B cells, CD4^+^ T cells, and monocytes in the low-risk group were higher. At the same time, the infiltration levels of M2 macrophages and myeloid dendritic cells in the high-risk group were higher. Two-factor survival analysis combining the immune cell scores and risk scores showed that UM patients with low risk scores and high immune cell scores showed better OS, while patients with high risk scores and low immune cell scores showed worse OS. Therefore, this six-gene signature may provide potential targets for immunotherapy of UM.

One of the important clinical characteristics of UM is the possibility of metastasis in the early stages of tumor development. However, the current traditional clinicopathological staging has limited predictive power for the prognosis of UM (38). Therefore, new therapeutic targets and immunomodulatory methods are urgently needed to improve the therapeutic effect and prognosis of patients with UM (39). Some studies have shown that microRNAs (miRNAs) such as miR-195, miR-224, miR-365a, miR-365b, miR-452, miR-4709, miR-7702, miR-513c, miR-873, miR-506-514 cluster, miR-592, and miR-199a are related to the OS of UM patients (40, 41). Meanwhile, some changes in the miRNA expression may be used to identify metastatic UM. Some changes in the miRNA expression in metastatic UM may be potential therapeutic targets (42). In addition, m6A RNA methylation regulators could also be used to assess the prognosis of patients with UM (43). As an effective tumor suppressor gene, *BRCA1*-associated protein 1 (*BAP1*) is related to the occurrence of some malignant tumors such as UM (44). Studies have shown that loss of expression of nBAP1 is an important biomarker for the progression and prognosis of UM (45). Therefore, *BAP1* immunohistochemical staining can be used to assess the risk of metastasis for UM (46). Besides, the elevation of serum markers such as LDH and alkaline phosphatase (ALP) is associated with shorter PFS and OS in UM patients. The expression changes of *IDO1* are related to the role of immune infiltrating cells in UM (47). The high expression of the long non-coding RNA (lncRNA) MIR155 host gene is associated with poor OS in UM patients and can be used to predict the efficacy of immune checkpoint inhibitor therapy (48). In this study, we constructed a new six-gene signature related to cancer hallmarks (including glycolysis and immune response). The survival analysis results confirmed that this six-gene signature, including *ARPC1B*, *BTBD6*, *GUSB*, *KRTCAP2*, *RHBDD3*, and *SLC39A4*, has good prognostic value for UM patients. It is worth mentioning that recent studies have established some prognostic gene signatures for UM as a supplement to traditional pathological staging. For example, a 10-gene signature established by Luo et al. showed excellent survival predictive ability for UM patients (23). We considered that a comparison of the survival predictive abilities of previously reported gene signatures and the six-gene signature will help to further explore the prognostic values of these gene signatures. Therefore, we compared the survival predictive abilities between the six-gene signature constructed in this study and the 10-gene signature previously reported by Luo et al. (23). The results showed that the two gene signatures had excellent survival predictive abilities in UM. The advantage of the six-gene signature is that it had fewer genes. In addition, although Kaplan–Meier analysis indicates that the six-gene signature showed certain predictive ability in both SM and HCC, the ROC analysis suggested that the prognostic value of the six-gene signature in UM was much better than that in SM and HCC.

In addition, KEGG analysis indicated that the enrichment pathways of DEGs between the high-risk and low-risk groups included phagosome, Epstein–Barr virus infection, cell adhesion molecules, human T-cell leukemia virus 1 infection, human cytomegalovirus infection, cytokine–cytokine receptor interaction, tuberculosis, *S. aureus* infection, antigen processing and presentation, and Th1 and Th2 cell differentiation, suggesting that the role of the immune microenvironment in UM is worthy of further study. As is known, immunotherapy has become a hotspot in the field of tumor therapy in recent years. Exploration of the molecular mechanisms related to immune response in UM is expected to provide new methods to improve the effect of immunotherapy.

Previous studies have shown that *ARPC1B* is associated with T- and B-cell immunodeficiency. *ARPC1B* can be used not only as a predictive biomarker for assessing the sensitivity of UM to radiotherapy but also as a prognostic biomarker for oral squamous cell carcinoma (49, 50). *GUSB* participates in molecular pathways such as the innate immune system and can be used as a biomarker to predict lymph node metastasis in patients with early cervical cancer (51). *SLC39A4* can be used as a prognostic marker for gastric cancer and non-small cell lung cancer (52, 53). In addition, there are few studies on the molecular mechanisms of *BTBD6*, *KRTCAP2*, and *RHBDD3* in UM, and their potential roles are worth exploring.

This study has some limitations. Firstly, because databases such as GEO and Genotype-Tissue Expression (GTEx) lack normal uveal transcriptome data, the cell distribution and expression levels of the prognostic gene markers in tumor tissues and adjacent normal tissues have not been explored further. Moreover, the robustness and the clinical availability of this six-gene signature need to be further verified in prospective trials. In addition, biological experimental research needs to be carried out to clarify the biological mechanism between the six-gene signature in UM and cancer hallmarks such as glycolysis and immune response.

## Conclusion

In conclusion, this study constructed a new six-gene signature (including *ARPC1B*, *BTBD6*, *GUSB*, *KRTCAP2*, *RHBDD3*, and *SLC39A4*) related to glycolysis and immune response from patients with UM. Besides, a nomogram based on the six-gene signature was constructed for clinical practice. This prognostic six-gene signature can not only be used as a prognostic biomarker to help clinicians develop more personalized treatments for patients with UM but also provides a potential therapeutic target for UM patients.

## Data Availability Statement

The datasets presented in this study can be found in online repositories. The names of the repository/repositories and accession number(s) can be found in the article/**Supplementary Material**.

## Author Contributions

WL designed the study and revised the manuscript. JL collected and analyzed the data. JJL interpreted the data and drafted the manuscript. All authors have read and approved the final manuscript. JL and JJL contributed equally to this work and are co-first authors. All authors contributed to the article and approved the submitted version.

## Conflict of Interest

The authors declare that the research was conducted in the absence of any commercial or financial relationships that could be construed as a potential conflict of interest.

## Publisher’s Note

All claims expressed in this article are solely those of the authors and do not necessarily represent those of their affiliated organizations, or those of the publisher, the editors and the reviewers. Any product that may be evaluated in this article, or claim that may be made by its manufacturer, is not guaranteed or endorsed by the publisher.
