# Supplementary material for: A Comprehensive Prognostic and Immunological Analysis of a Six-Gene Signature Associated With Glycolysis and Immune Response in Uveal Melanoma
Source: Front Immunol. 2021 Sep 22;12:738068. doi: 10.3389/fimmu.2021.738068 (PMC8494389; doi:10.3389/fimmu.2021.738068)
Supplement: Supplementary file 1 [file DataSheet_1.docx]

**Supplementary Figures**


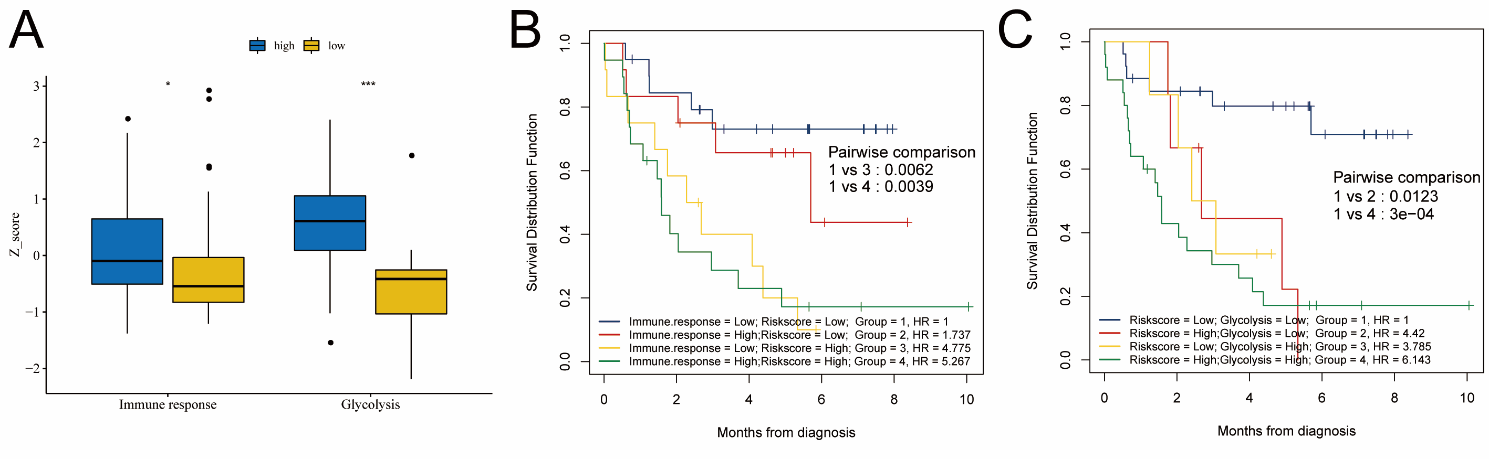


**Figure S1: Two-factor survival analysis combining cancer hallmarks and risk scores in the UM cohort from GSE22138.** **(A)** The Z-socres of immune response and glycolysis in the high-risk group were higher than those in the low-risk group. **(B)** Two-factor survival analysis combining immune response and risk scores showed that high immune prevention & high risk scores predicted a worse prognosis. **(C)** Two-factor survival analysis combining glycolysis and risk scores showed that high glycolysis & high risk scores predicted a worse prognosis.


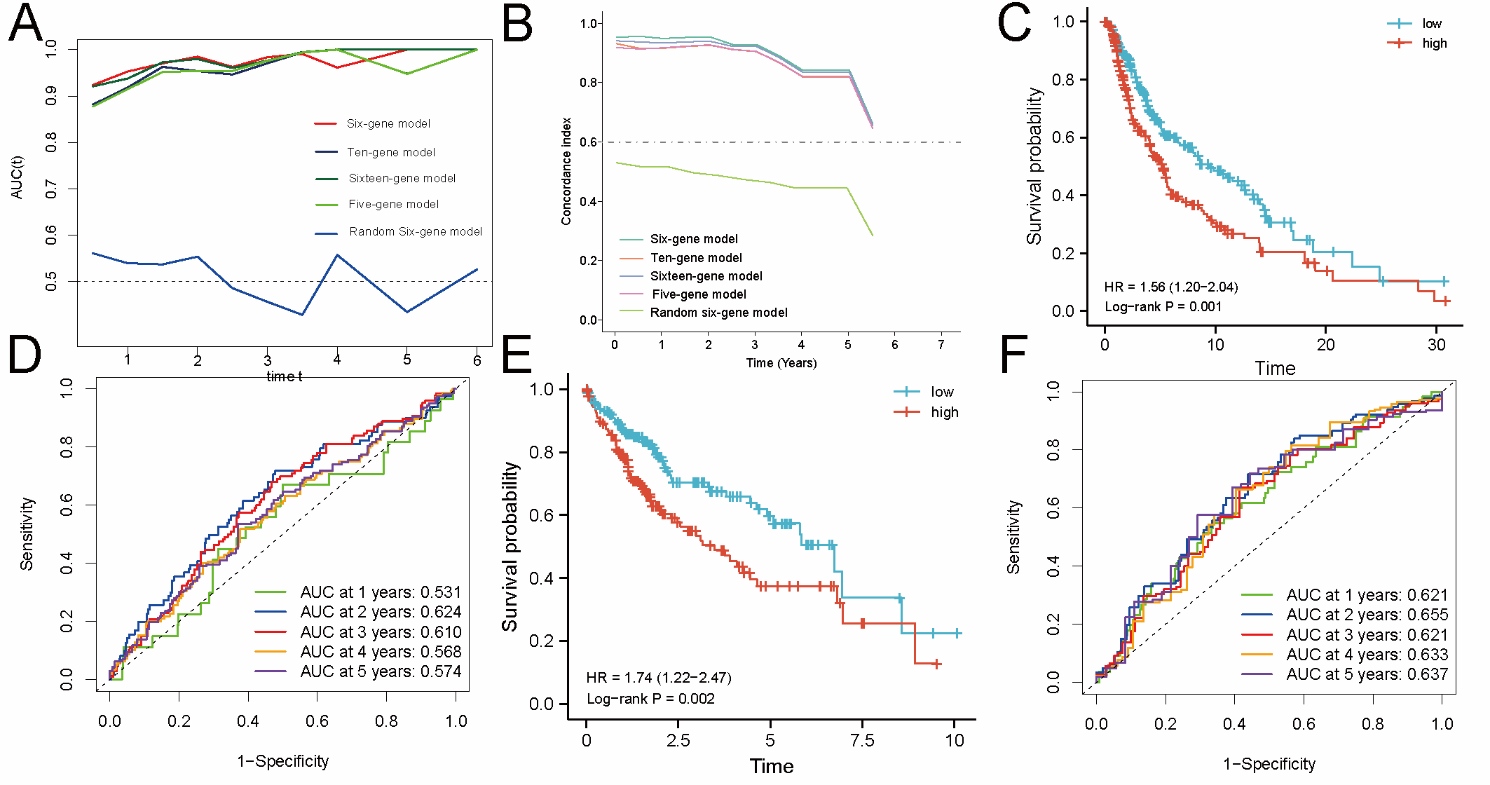


**Figure S2:** **The prognostic performance of the six-gene signature in UM, skin melanoma and hepatocellular carcinoma.** **(A-B)** The predictive abilities of various gene signatures in UM were compared. Five gene signatures were defined as follows: the six-gene signature was constructed in this study; the ten-gene signature (SIRT3, HMCES, SLC44A3, TCTN1, STPG1, POMGNT2, RNF208, ANXA2P2, ULBP1, and CA12) was constructed by Luo Huan et al.; the sixteen-gene signature was constructed by combining the six-gene signature and ten-gene signature; the five-gene signature (ANXA2P2, CA12, HMCES, SIRT3, and SLC44A3) was constructed by performing stepwise multi-factor COX regression analysis on the ten-gene signature; the random six-gene signature was constructed by randomly selecting six genes from the UM transcriptome data. The tROC analysis and C-index analysis indicated that the six-gene signature, ten-gene signature, sixteen-gene signature and five-gene signature showed good survival prediction abilities (AUC>0.85). And the prediction results of these four gene signatures were consistent with the survival results actually observed in UM. In addition, the random six-gene signature failed to predict survival in UM (AUC<0.57). **(C-D)** Kaplan-Meier and ROC curves showed that the six-gene signature had low predictive power in skin melanoma (AUC<0.63). **(E-F)** Kaplan-Meier and ROC curves showed that the six-gene signature had low predictive power in hepatocellular carcinoma (AUC<0.66). tROC, time-dependent receiver operating characteristics. C-index, consistency index.


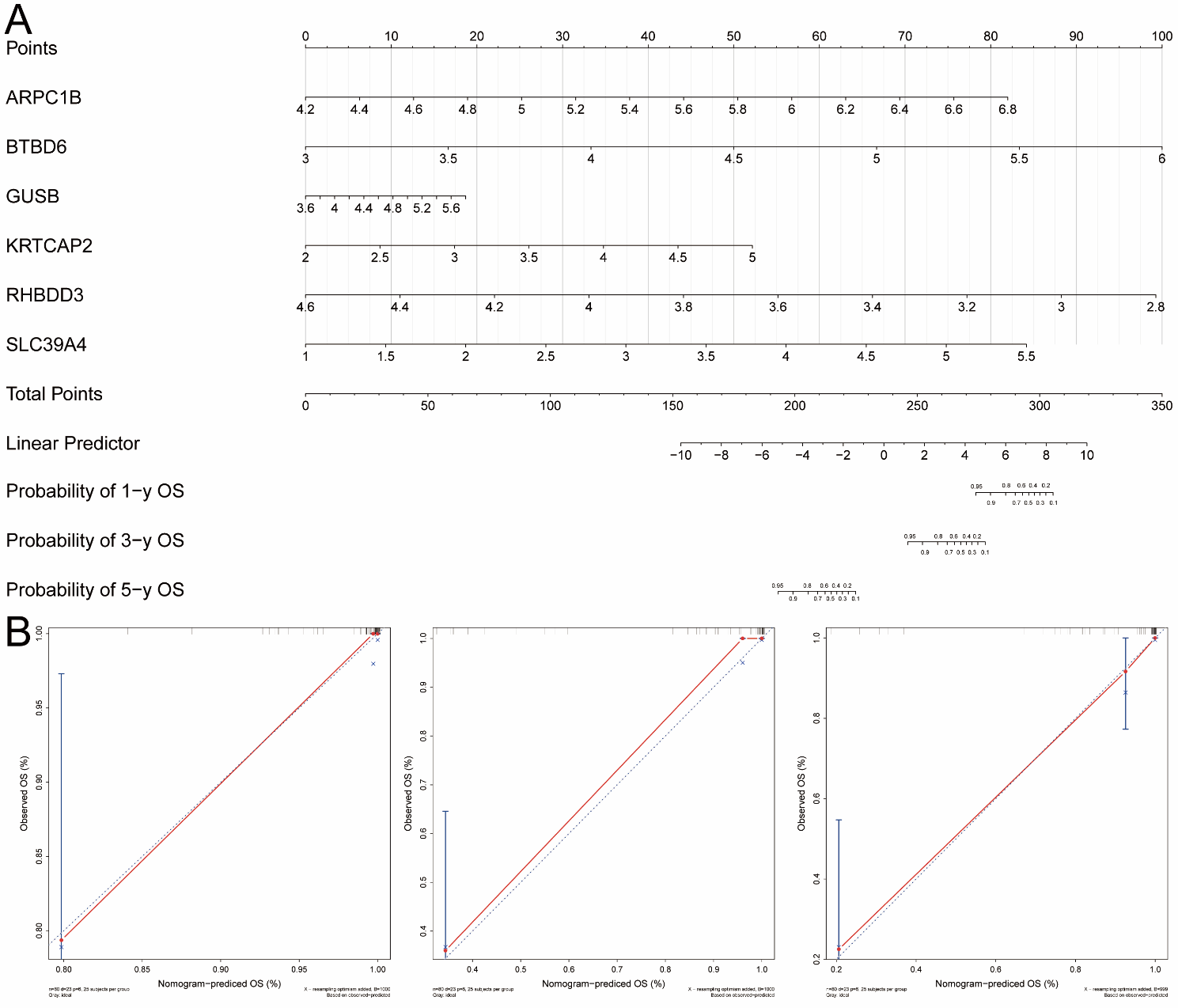


**Figure S3: Construct a nomogram based on six-gene signature for overall survival prediction. (A)** A nomogram based on the expression profiles of the six-gene signature. **(B)** The calibration chart showed that the predicted 1- 3-, and 5-year survival probabilities were in good agreement with actual observations.


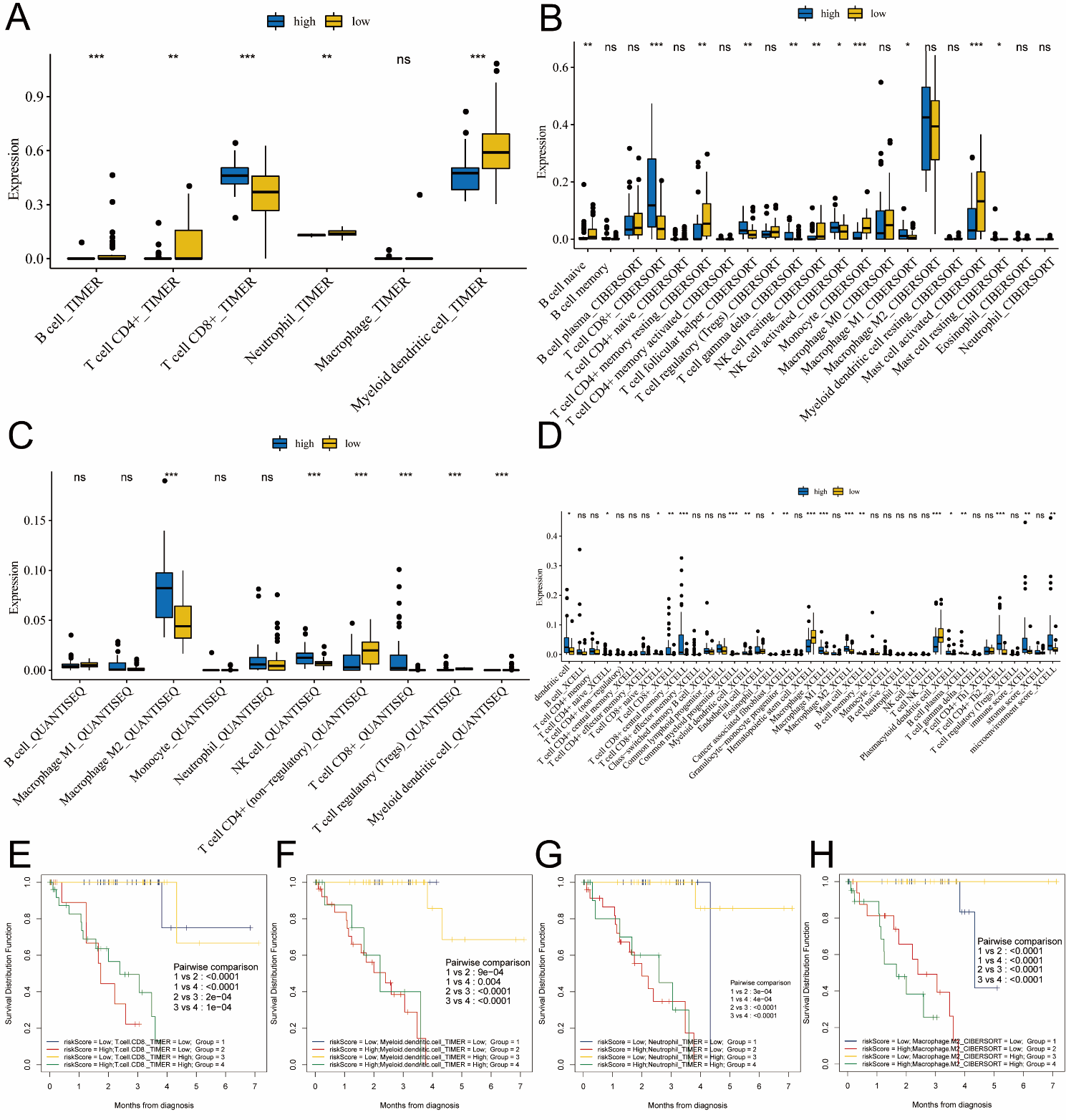


**Figure S4: Correlation analysis between risk score and degrees of immune cell infiltration.** **(A-D)** TIMER, CIBERSORT, QUANTISEQ, XCELL were used to evaluate the correlations between the risk scores and the degrees of immune cell infiltration. **(E-H)** Two-factor survival analysis combining immune cell scores and risk scores showed that patients with low immune cell scores (including T cell CD8, myeloid dendritic cell, neutrophil or macrophage) and high risk scores had poor prognosis.
